# The Impact of the Cellular Environment and Aging on Modeling Alzheimer's Disease in 3D Cell Culture Models

**DOI:** 10.1002/advs.202205037

**Published:** 2023-01-15

**Authors:** Matthias Hebisch, Stefanie Klostermeier, Katharina Wolf, Aldo R. Boccaccini, Stephan E. Wolf, Rudolph E. Tanzi, Doo Yeon Kim

**Affiliations:** ^1^ Genetics and Aging Research Unit McCance Center for Brain health, MassGeneral Institute for Neurodegenerative Disease Massachusetts General Hospital Harvard Medical School Charlestown MA 02129 USA; ^2^ Institute of Medical Physics Friedrich‐Alexander Universität Erlangen‐Nürnberg 91052 Erlangen Germany; ^3^ Max‐Planck‐Zentrum für Physik und Medizin 91054 Erlangen Germany; ^4^ Department of Medicine 1 Friedrich‐Alexander‐Universität Erlangen‐Nürnberg 91054 Erlangen Germany; ^5^ Institute of Biomaterials Department of Materials Science and Engineering Friedrich‐Alexander‐Universität Erlangen‐Nürnberg 91058 Erlangen Germany; ^6^ Institute of Glass and Ceramics Department of Materials Science and Engineering Friedrich‐Alexander‐Universität Erlangen‐Nürnberg 91058 Erlangen Germany

**Keywords:** Alzheimer, hydrogel, neurodegeneration, nonclassical crystallization, synthetic extracellular matrix

## Abstract

Creating a cellular model of Alzheimer's disease (AD) that accurately recapitulates disease pathology has been a longstanding challenge. Recent studies showed that human AD neural cells, integrated into three‐dimensional (3D) hydrogel matrix, display key features of AD neuropathology. Like in the human brain, the extracellular matrix (ECM) plays a critical role in determining the rate of neuropathogenesis in hydrogel‐based 3D cellular models. Aging, the greatest risk factor for AD, significantly alters brain ECM properties. Therefore, it is important to understand how age‐associated changes in ECM affect accumulation of pathogenic molecules, neuroinflammation, and neurodegeneration in AD patients and in vitro models. In this review, mechanistic hypotheses is presented to address the impact of the ECM properties and their changes with aging on AD and AD‐related dementias. Altered ECM characteristics in aged brains, including matrix stiffness, pore size, and composition, will contribute to disease pathogenesis by modulating the accumulation, propagation, and spreading of pathogenic molecules of AD. Emerging hydrogel‐based disease models with differing ECM properties provide an exciting opportunity to study the impact of brain ECM aging on AD pathogenesis, providing novel mechanistic insights. Understanding the role of ECM aging in AD pathogenesis should also improve modeling AD in 3D hydrogel systems.

## Graphical abstract

Emerging hydrogel‐based 3D neural cell culture models provide an exciting opportunity to study the impact of brain extracellular matrix (ECM) and its aging on Alzheimer's disease (AD) pathogenesis. Altered ECM characteristics in aged brains contribute to disease pathogenesis by modulating the accumulation, propagation, and spreading of pathogenic molecules of AD. Understanding the pathogenic role of ECM aging will also improve modeling AD in a dish.

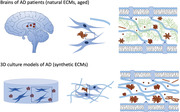

## 1 Introduction

The ECM‐ encompasses the structural and functional components surrounding cells in 3D space. It provides mechanical stability, adhesion points, and a reservoir for growth factors. Furthermore, it offers directional cues for cell migration and polarity. In addition, ECM is essential for water storage and compartmentalization. The full complexity of ECM found in tissues is usually not required to cultivate single cell types in vitro. Yet, failing to provide appropriate ECM structures can cause many typical cell culture artifacts like morphological differences, altered proliferation, and intracellular signaling through mechanotransduction, as well as changes to global histone acetylation affecting gene expression.^[^ ^1^, ^2^, ^3^, ^4^ ^]^ Beyond supporting normal physiological function, ECM alterations can play a role in various pathologies. For example, the ECM can contribute to disease in the human brain by regulating the accumulation, propagation, and spreading of pathogenic molecules.

**Figure 1:**
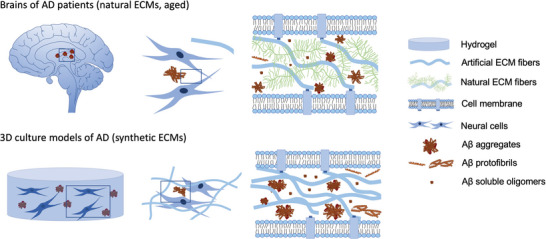
Biological extracellular matrix of the human brain compared to a 3D cell culture system mimicking the biological extracellular matrix. The extracellular matrix is a complex assembly of numerous proteins and polysaccharides building an elaborate meshwork of tissue‐specific composition. The main components such as fibrous structural proteins (e.g., collagens, laminins, or fibronectin), specialized proteins (e.g., growth factors, small integrin‐binding glycoproteins) and proteoglycans undergo a constant remodeling process. ECM replacements offer similar hydrogel structures but differ in the exact composition and architecture.

In AD, soluble amyloid‐*β* (A*β*) peptides accumulate and aggregate in the extracellular space to form so‐called A*β* plaques.^[^ ^5^, ^6^ ^]^ Modeling amyloid plaque pathology in vitro has been a longstanding challenge in the AD field, especially using conventional two‐dimensional (2D) culture systems. In contrast, mouse models show robust amyloid plaque deposition within a few months (e.g., APP23, 5XFAD).^[^ ^7^, ^8^ ^]^ Starting from this discrepancy, we previously reported that a 3D gel culture system enables local accumulation of secreted A*β* species sufficient to induce robust aggregation of A*β*, which is similar to A*β* plaques in the brains of AD patients (**Figure** **1**). Importantly, we found that accumulation of A*β* in 3D gels induced accumulation and aggregation of hyperphosphorylated tau protein (neurofibrillary tangle, NFT).^[^ ^9^, ^10^ ^]^ Our results highlight the importance of 3D ECM in mediating or accelerating disease pathogenesis. Since then, other studies have also confirmed the indispensability of 3D ECM structures on pathogenic A*β* accumulation and A*β*‐induced pathological cascade in 3D cellular models, including 3D brain organoid models and neural stem cell‐derived spheroids.^[^ ^10^, ^11^, ^12^, ^13^, ^14^, ^15^, ^16^, ^17^, ^18^ ^]^

Despite these successes, the mechanisms governing the accumulation and aggregation of A*β* species, in 3D matrices are poorly understood. The 3D matrices would also affect the diffusion and accumulation of other pathogenic molecules associated with AD, including oligomeric tau species, cytokine/chemokines, and even viruses. The heterogeneous nature of 3D matrices used in 3D culture systems makes it difficult to dissect the contributions of single matrix components and their physical properties on AD pathology. Studying the impact of 3D matrices in AD cellular models would also contribute to understanding how brain ECM and aging affect the accumulation and aggregation of pathogenic molecules in AD patients. Indeed, Jucker's and Fändrich's laboratories analyzed the structure of pure, isolated A*β* fibrils from AD patients using cryo‐electron microscopy (cryo‐EM).^[^ ^19^ ^]^ Unlike the left‐handed fibrils observed in A*β* fibrils from the in vitro aggregation of synthetic A*β* peptides monomers, the new AD‐patient‐derived A*β* fibrils showed dominantly right‐handed single, double, and triple‐helical fibrils in meningeal amyloid, which is the opposite of solution‐aggregated A*β* fibril structures. The presence of brain ECM may explain these discrepancies in A*β* fibril structures.

This review will discuss the physiologic and pathogenic impact of ECM in 3D human neural models and brains of AD patients. We will comprehensively address how ECM characteristics, including matrix stiffness, density, pore size, and composition, regulate neural differentiation and network formation and contribute to the accumulation, aggregation, and propagation of pathogenic molecules of AD, including A*β* oligomers, soluble tau species, and other soluble molecules known to contribute to AD pathogenesis. Finally, we propose that these understudied parameters play an important role in modeling AD pathogenesis in a dish and possibly in aged human brains by regulating the generation and propagation of pathogenic molecules of the disease.

## 2 Mimicking the Structures and Function of Natural ECMs with Various 3D Hydrogels

In most tissues, the ECM spans a complex 3D fibrous mesh that consists of fibers and pores. This meshwork comprises collagen and elastic fibers embedded in a highly hydrated gel of glycoproteins, glycosaminoglycans, and proteoglycans.^[^ ^20^ ^]^ It is instrumental in structuring tissues, regulating secreted factor gradients as a buffer for water and various solvents, disposing of metabolic waste, and absorbing compressive and tensile stresses. Accordingly, the ECM is highly complex and consists of numerous proteins, such as collagens, fibronectins, laminins, tenascins, and proteoglycans.^[^ ^21^, ^22^ ^]^

**Figure 2:**
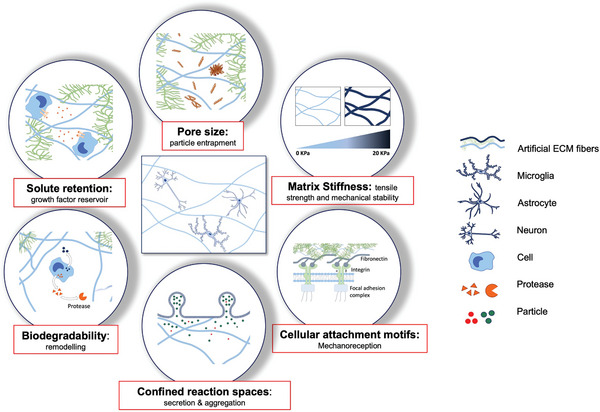
Altered 3D ECM matrix properties regulates accumulation and aggregation of pathogenic molecules in brain diseases. The changes in ECM stiffness and pore size modulate diffusion and aggregation of pathogenic molecules, including pathogenic A*β* and tau oligomers. Aging‐associated oxidation of matrix protein can alter cellular attachment motifs that activate intracellular cell signaling cascades that lead to inflammatory response and the altered neural‐glial crosstalk. Changes in ECM composition also triggers the binding and pathogenic aggregation of A*β* species. Changes in ECM characteristics can also adjust the balance among accumulation (aggregation), diffusion, and degradation of pathogenic particles, including A*β*, tau oligomers, cytokines, exosomes, and large viral particles implicated in the pathogenic cascade of brain disorders.

Generally, five major parameters govern ECM properties: Stiffness, pore size, cellular attachment motifs, biodegradability, and solute retention^[^ ^23^, ^24^, ^25^, ^26^, ^27^ ^]^ (**Figure** **2**). Stiffness and pore size are partly interlinked in an anti‐proportional relationship. A higher amount of matrix building blocks in the same volume of space increases the stiffness while decreasing the average pore size. Nonetheless, fine‐tuning the exact micro‐ to nanoscale topology of the matrix is possible by adjusting the monomer concentration.^[^ ^28^ ^]^ Varying the ratio of monomers and crosslinkers results in either thick polymer strands with large pores or a finely‐spun meshwork of polymer filaments and tiny pores.^[^ ^29^ ^]^ For instance, hydrogels mimicking the mechanical properties of brain have been developed recently to support, e.g., surgical exercises.^[^ ^30^ ^]^ The gel stiffness regulates proliferation, migration, and differentiation in various cell types, including neurons, astrocytes, and oligodendrocyte precursors.^[^ ^31^, ^32^, ^33^, ^34^ ^]^ In general, cells appear to thrive most in matrices that mimic the stiffness of their natural niche.

Cells actively interact with the surrounding ECM and vice versa. Mechanical stiffening of the ECM can lead to intracellular Rho activation. Cells react by cytoskeletal reorganization through focal adhesion maturation and increased contractile force generation.^[^ ^35^ ^]^ Increasing matrix traction and compaction then elevates ECM stiffness further.^[^ ^36^ ^]^ This positive feedback loop is counterbalanced by negative regulation via mechanically induced matrix remodeling.^[^ ^37^ ^]^ Since these features are tissue‐specific, 3D cell culture matrices need to be designed to mimic the key features of the ECM for each application – but in most cases, these are not yet known.

Many studies demonstrate that cultivating cells on mismatched ECM can detrimentally influence cellular morphology, metabolism, and signaling.^[^ ^1^, ^2^, ^3^, ^4^ ^]^ This critical issue of “mismatching” matrices has been demonstrated.^[^ ^38^ ^]^ Even more reductionist approaches revealed that single‐cell types often require just one specific set of binding motifs to show in vivo‐like properties. These findings enabled significant strides toward xeno‐free, chemically defined cell culture conditions, and, as a result, synthetic functionalized 3D matrices were developed. The downside of this development is the loss of many vital features of the natural ECM, such as in vivo‐like porosity, permeability, mechanical characteristics, solute retention, and nanoscale morphology, which may be critical for recapitulating disease conditions in a dish.^[^ ^39^ ^]^

Different gel materials imitate the mechanical, compositional, and structural conditions of the extracellular milieus of tissues, including hydroxyapatite ceramics, fibrillar sponges, glass‐based substances, or polymeric hydrogels. Hydrogels are particularly suitable for imitating the ECM, consisting of a soft, fibrillar, and diffusive fiber network. They store large amounts of water and provide a high diffusivity to the solute due to their porous structure and can be functionalized with cell adhesion sites. Hydrogels can be classified into the following three classes according to their origin: Biological (Matrigel,^[^ ^40^, ^41^ ^]^ collagen I,^[^ ^42^, ^43^, ^44^ ^]^ alginate^[^ ^45^, ^46^ ^]^), synthetic (polyacrylamide, polyethylene glycol (PEG)^[^ ^12^, ^47^ ^]^), or hybrid (hyaluronic acid, polypeptides, silk, fibrin) materials^[^ ^48^, ^49^, ^50^, ^51^, ^52^, ^53^, ^54^ ^]^ (**Table** **1**). Alginate‐based ionotropic hydrogels or synthetic hydrogels like PEG offer superior standardization compared to cell culture‐derived options.^[^ ^45^ ^]^ Fully synthetic polyethylene glycol (PEG)‐based gels are often synthesized for specific applications and can be functionalized as required, offering biocompatibility and a high control of reaction kinetics, e.g., during photopolymerization and ‐degradation.^[^ ^55^ ^]^ Hydrogels made from polymeric materials can store large amounts of water and offer a porous, soft, 3D matrix. Especially the fast but gentle polymerization conditions of these natural or synthetic hydrogels are crucial for the embedded cells and generally suitable for simulating neural ECM.^[^ ^50^ ^]^

**Table 1:** Overview of common hydrogels for 3D cell‐culture models

Matrix	Type	Material	Advantages	Disadvantages	Used for	Refs.
Matrigel	Cell culture‐derived	ECM and membrane fraction	Easy to use	Animal‐derived	Coating	[40, 41]
			Broad compatibility	No tunable material characteristics	3D cell culture	
			Widely accepted standard	Insufficiently characterized		
				Batch to batch variations		
Collagen I	Animal‐derived	Bovine collagen type I	Offers only one integrin binding site (Arg‐Gly‐Asp)	(Animal‐derived)	3D cell culture	[42, 43, 44]
	Human‐derived	Human collagen type I		No tunable material characteristics		
				No crosslinked functional groups such as IKVAV		
				Batch to batch variations		
Alginate	Plant‐derived	Brown algae polymer	Animal‐free	Contains no binding motifs for animal cells	3D cell culture	[45, 46]
			Gentle polymerization by physiological levels of calcium			
			Biophysical properties can be tuned			
PEG	Synthetic	Polyethylenglycol	Xeno‐free	No isolated tunability of viscosity and elastic stiffness	3D cell culture	[12, 47]
			Biophysical properties can be tuned			
			Batch to batch consistency			
			Crosslinkable functional groups			
Silk	Animal‐derived	Silkworm cocoon polymer	Scaffold material	Contains no binding motifs for animal cells	3D cell culture	[51, 52]
			Compatible with other polymers, e.g., collagen, Matrigel	No tunable material characteristics		
			Retention of growth factors or other functionalization			
Fibrin	Synthetic	Blood clotting polymer	Xeno‐free formulations available	Rapid degradation	3D cell culture	[53, 54]
			Gel stability	No tunable material characteristics	Implants	
			Biocompatibility			

Interestingly, the presence or absence of attachment motifs in hydrogels has an outsized effect on the embedded cells. Alginate, for example, displays good biocompatibility, yet it lacks cell and protein adsorption due to the missing adherence motifs.^[^ ^49^ ^]^ Chemical modifications of bioinert hydrogels were developed to functionalize them with different amino acid sequences like RGD (Arg‐Gly‐Asp), YIGSR (Tyr‐Ile‐Gly‐Ser‐Arg), and IKVAV (Ile‐Lys‐Val‐Ala‐Val) to promote cell attachment.^[^ ^45^, ^56^, ^57^, ^58^ ^]^ In 2015, Dr. Searson's laboratory demonstrated the interplay between the above‐mentioned parameters using primary human astrocytes in collagen, hyaluronic acid (HA), and Matrigel hydrogels by systematically varying gel stiffness and composition to achieve complex and nonreactive astrocytic phenotypes.^[^ ^59^ ^]^ The same concept of functionalization motifs can also be applied to add target sites for cellular proteases to enable matrix remodeling. Matrix degradability is also important for culture parameters like cell proliferation and migration. Studies from the Lutolf and Chen laboratories have demonstrated that cell motility and proliferation in a 3D volume require the matrix to be degradable.^[^ ^60^, ^61^ ^]^ But in extreme cases, very stiff matrices can still trap cells in place.^[^ ^60^, ^61^ ^]^ Madhusudanan and colleagues provide a comprehensive overview of matrix systems and their properties used to cultivate neural cells.^[^ ^62^ ^]^ Table 1 briefly summarizes natural and synthetic ECM matrix materials suitable for 3D matrix cell culture models.

Brain organoid models might be the only model that can recapitulate the most complex brain architecture in a dish and possibly brain ECM environment, although it has not been fully characterized. Since neural stem cells have been shown to secrete their own matrix, it can be assumed to be largely endogenous.^[^ ^60^, ^63^ ^]^ Nonetheless, widely used protocols for cerebral organoid generation call for embedding of the immature organoid in a solidified Matrigel drop, which likely affects standardization.^[^ ^64^, ^65^ ^]^ Neural differentiation and morphogenesis are affected by the Matrigel environment, and it is not known to what degree Matrigel components linger in or penetrate into mature organoids. And indeed, first studies from the Knoblich lab demonstrated that the basement membrane of the cortical plate is dependent on Matrigel supplementation that cannot be replaced by laminin, entactin, collagen, or a combination thereof.^[^ ^66^ ^]^ It should be noted that not all organoid methods rely on exogenous matrix components, for instance protocols from the Pasça group.^[^ ^67^, ^68^ ^]^ Further comparative studies will be needed to determine which 3D brain organoid and/or spheroid models are advantageous for mimicking human brain ECM. But brain organoids/spheroids typically recapitulate early embryonic brain development. Whether modeling of aged brain ECM is possible in those setups is not yet clear. It follows that there is potential for comparative analyses between these different protocols to define the role of ECM components in development. While not yet conclusive, these examples illustrate the potential for experimental probing of the ECM in 3D organoid systems.

Matrigel, a solubilized basement membrane extract from the Engelbreth–Holm–Swarm (EHS) mouse sarcoma, has been widely used in 2D and 3D human brain cell culture models. However, the composition of Matrigel has not been fully defined, and its “lot‐to‐lot” variability may affect the experiment's reproducibility.^[^ ^40^ ^]^ Proteomic analyses reveal batch‐to‐batch inconsistencies, spotting, e.g., ILG1 and EGF in significant (but also highly variable) quantities in a subset of batches.^[^ ^40^, ^41^, ^69^, ^70^ ^]^ Moreover, mechanical properties also vary even within batches after accounting for different testing methods and external testing conditions.^[^ ^71^, ^72^, ^73^, ^74^, ^75^ ^]^ This results on a microscale variability of measured elastic moduli and viscosities.^[^ ^75^ ^]^ In addition, the presence of xenogeneic ECM contaminants (e.g., lactate dehydrogenase‐elevating virus) makes it challenging to use Matrigel for preclinical drug testing.^[^ ^76^, ^77^ ^]^ Because of those limitations, a more innovative replacement matrix would be highly desirable for the next‐generation 3D culture models.

## 3 A*β* Aggregation in Solutions and 3D Hydrogels

Studying amyloid pathology in 3D systems paves the way toward a better understanding of A*β* aggregation in brain‐like ECM conditions, compared to the conventional 2D cell culture system. Despite tremendous efforts, the mechanisms of A*β* aggregation in 3D brain ECM are not fully known, especially since recent cryo‐EM studies indicated that commonly used sample amplification techniques might introduce catastrophic structural artifacts.^[^ ^19^ ^]^ Here, we provide a concise overview of theories of A*β* aggregation and how this is affected by 3D matrices including brains and 3D cellular models.

Since initial A*β* clusters are likely weak agglomerates, bound together by weak physical interactions such as dipole and van der Waals forces, they can still dissociate into monomeric forms. Agglomerated proteins can retain their folding state initially exhibited in solution, and mixed agglomerates are composed of proteins of different types and/or folding states.^[^ ^78^ ^]^ Over time, agglomerates age, densify and conformationally reorganize, e.g., developing extended *β*‐sheet motifs. For instance, the aging of A*β* agglomerates yield one‐stranded filaments.^[^ ^79^, ^80^, ^81^, ^82^, ^83^, ^84^, ^85^, ^86^, ^87^, ^88^ ^]^ These first fibril‐like species can then grow further through recruitment of intrinsically disordered peptides, which simultaneously undergo refolding. Although this is a complex starting scenario, amyloid fibrillogenesis can be often described well in terms of classical nucleation theory (CNT).^[^ ^89^, ^90^, ^91^, ^92^, ^93^, ^94^, ^95^ ^]^

The theoretical framework of CNT describes the separation of a new phase or particle from a mother phase as a one‐step process, and it rests on two assumptions. First, it presumes that the internal structure of the nucleus is identical to that of the later bulk phase. Second, it postulates that growth only occurs by attachment of monomers, i.e., single ions or molecules (here proteins or peptides). These assumptions provide a framework for a general physical description of nucleation and growth processes. However, more intricate processes—such as clustering and aggregation due to weak solute interactions—are not considered in CNT.^[^ ^96^, ^97^ ^]^ Today, nonclassical routes—involving precritical clustering—have been identified in a remarkable number of systems,^[^ ^96^ ^]^ including amyloid fibrillogenesis.^[^ ^98^ ^]^ For instance, Yamamoto et al. showed in the peptide model system of insulin B chain peptides that the attachment of globular protein aggregates feeds amyloid fibrogenesis.^[^ ^99^ ^]^ Moreover, van Driessche and co‐workers demonstrated that fibrillogenesis is driven by oriented attachment of pre‐formed and near‐to crystalline protein clusters.^[^ ^100^ ^]^

How do these newly identified nonclassical traits of amyloid fibrillogenesis impact the further development of new 3D cellular model systems composed of synthetic and natural ECMs? They highlight that protein refolding, aggregation, and nonclassical crystallization processes are crucial to amyloid fibrillogenesis. All of these processes are markedly susceptible to solution composition. With this, it becomes apparent that we have to exert strict control over the fluid and 3D gel composition to strengthen our control, reproducibility, and comparability of amyloid fibrillogenesis in cell culture systems: salinity, buffer composition, and organic cosolutes have to be fixed to allow comparability across model systems. This is because protein folding, as well as colloid–colloid (such as protein–protein) interactions, are strongly affected by these parameters. Even a higher concentration of charged cosolutes can already, due to electrostatic repulsions effects, enhance aggregation. Larger cosolutes, such as (bio)polymers can also have dramatic colloid‐chemical effects, leading to aggregation or phase separation, e.g., by depletion destabilization effects. Thus, precise knowledge of the biological fluid composition is a precondition for a thorough understanding of initial protein A*β* agglomeration in particular and in amyloid fibrillogenesis in general.

Indeed, van Driessche and colleagues reported that the solution composition (e.g., salinity or other polymeric cosolutes such as PEG) impacts fibrillogenesis.^[^ ^100^ ^]^ In line with this report, A*β*42 fibrillogenesis is highly dependent on the solution's salinity, i.e., the solution's ionic strength.^[^ ^90^, ^101^, ^102^, ^103^ ^]^ Therefore, the composition of the ECM (or employed buffers) has to be meticulously controlled and reported to allow for comparability and reproducibility. These considerations also imply that we should strive for realistic biomimetic model systems, with solution compositions that are fully comparable to the A*β* aggregation in brains of AD patients.

## 4 Effects of Fluid Characteristics on A*β* Accumulation and Aggregation

Besides the solid components of the extracellular space, there is also an extracellular fluid composed mainly of water with dissolved electrolytes, metabolites, and proteins (e.g., hormones, enzymes, and neurotransmitters). The fluid‐containing brain compartments, i.e., the ventricles, interstitial space, and vascular space, contain cerebrospinal fluid (CSF), interstitial fluid (ISF), and blood plasma, respectively.^[^ ^104^, ^105^ ^]^ The current model suggests an exchange of CSF and ISF via diffusion through the ependyma, there are no tight junctions between neuro endothelial cells.^[^ ^106^ ^]^ However, especially for large molecules, this diffusion‐driven exchange of CSF and ISF was estimated to be inefficient. In 2012, Iliff et al. proposed a new, Aquaporin‐4 (AQP‐4) driven paravascular pathway for the clearance of the brain's extracellular fluids denominated “glymphatic system” that is hypothesized to contribute to macromolecule clearance.^[^ ^105^, ^107^, ^108^ ^]^

Composition, flow, and clearance of CSF and ISF play an important role in developing neurodegenerative diseases.^[^ ^109^, ^110^ ^]^ Accumulation of A*β* and tau protein in the ISF leads to their oligomerization and aggregation resulting in synaptotoxicity and neurodegeneration leading to dementia in humans.^[^ ^111^, ^112^, ^113^ ^]^ Reducing the ISF/CSF flow would accelerate A*β* aggregation and AD pathogenesis.^[^ ^114^, ^115^ ^]^ Furthermore, the deletion of AQP4 was shown to promote A*β* accumulation in the brain parenchyma and trigger memory deficits in rodents.^[^ ^116^, ^117^ ^]^ Especially during sleep, clearance of ISF and CSF is significantly augmented compared to wakefulness because of an increase in interstitial space, which results in enhanced convective CSF‐ISF exchange.^[^ ^118^ ^]^ Thus, sleep deprivation was suggested to increase the amount of A*β* and tau aggregates in the brain, promoting AD.^[^ ^119^, ^120^ ^]^ It is not fully understood whether other solutes accumulating in the ISF in case of changes in composition, flow, or clearance, might introduce or influence tau and/or A*β* aggregation. Besides, there might be an impact of the brain's ECM on ISF/CSF flow and clearance: ECM constitutes a flow resistance, it might mediate hydrostatic and osmotic forces as it interacts with ions and proteins, and it is involved in structuring and stabilization of fluid‐filled compartments.^[^ ^121^, ^122^ ^]^

## 5 The Impact of 3D ECM on Recapitulating AD Pathogenic Cascades in 3D Human Cell Culture Systems

Classical 2D culture models of AD show some amyloid‐related pathological changes but lack plaque formation and robust neurofibrillary tangle (NFT) pathology (reviewed^[^ ^123^ ^]^). Occasional observations of such features are restricted to immunostainings of clumped neurons or rely on large quantities of synthetic A*β* in the culture medium.^[^ ^124^, ^125^ ^]^ Endogenously generated ECM in the neuronal clumps may provide 3D structures for small A*β* aggregates.^[^ ^124^ ^]^ Therefore, we hypothesized that A*β* secreted into the supernatant of 2D cultures would be too diluted to form aggregates and that any severe accumulation would be prevented by medium replacement. Thus, to limit the diffusibility of A*β* and enable local accumulation, we embedded our cultures in a 3D Matrigel (Corning, USA), enriched with brain extracellular matrix proteins, including laminin, collagen type IV, heparan sulfate proteoglycans, and entactin.^[^ ^10^, ^126^ ^]^

Supernatants of 3D cultures contained only trace amounts of soluble A*β* compared to identical 2D cultures, indicating that the gel matrix promoted A*β* retention. Indeed, within 6–12 weeks, 3D cultures generated amyloid plaques and a wide variety of oligomeric A*β* species.^[^ ^10^ ^]^ The deposited amyloid material was resistant to SDS dissolution and reacted to *β*‐fibril dyes (e.g., Thioflavin T, AmyloGlo). We could also show robust p‐tau pathology in western blot, immunohistochemistry, Gallyas staining, and electron microscopy analyses.^[^ ^10^ ^]^ Amyloid deposition and p‐tau pathology could be prevented by chemical inhibition of A*β* release from APP, showing that neither pathology is an artifact of 3D cultivation. Finally, we found that not the amount of released A*β* but the ratio between the 42 and 40 amino acid species is the most vital determinant of downstream pathology (e.g., NFT). We also showed that the ratio between pathogenic A*β*42 and nonpathogenic A*β*40 isoform, not total A*β* levels, determine tau pathology and neuronal death,^[^ ^9^ ^]^ which is consistent with the amyloid cascade hypothesis.^[^ ^6^ ^]^ This notion is further confirmed by the predominance of FAD mutations that decrease A*β*40 generation but do not increase A*β*42.^[^ ^127^ ^]^

3D culture conditions seem to accelerate AD pathology in human iPSC‐derived neural cellular models. Human iPSC‐derived neurons harboring FAD mutations do not consistently display tau pathology in 2D culture conditions, depending on the location of FAD mutations, APP, or PS1.^[^ ^128^ ^]^ However, 3D AD brain organoid models, encapsulated in 3D Matrigel, readily showed A*β* accumulation and tau pathology with iPSCs harboring APP or PS1 familial mutations.^[^ ^129^, ^130^, ^131^ ^]^ In addition, brain organoids with APOE4, a major risk factor for sporadic AD, also showed A*β* accumulation and A*β* ‐induced tau pathology, which was not feasible with conventional 2D culture models.^[^ ^16^, ^132^, ^133^ ^]^ These findings strongly support that 3D culture conditions have an advantage in accelerating A*β* accumulation and A*β*‐triggered tau pathology in cellular AD models.

The 3D ECM also accelerates the expression of adult 4‐repeat (4R) tau splice isoforms, which is critical for recapitulating tau pathology in AD and AD‐related diseases. Conceivably, the development of NFT pathology might depend on neuronal maturation, especially on the availability of 4‐repeat (4R) tau splice isoforms. Indeed, 3D cultivation conditions seem to improve neuronal development.^[^ ^64^, ^134^ ^]^ We demonstrated that 3D cultivation techniques significantly accelerate 4R tau isoform expression in our 3D AD cellular models based on immortalized human neural progenitor cells.^[^ ^9^, ^10^ ^]^ 3D ECM environments also accelerate 4R tau expression in human iPSC‐derived neuronal cultures, even though the adult brain 3R:4R tau ratio of 1:1 was not achieved.^[^ ^135^ ^]^ These underscore advantage of using 3D culture models with ECM in modeling Alzheimer's tau pathology.

## 6 The Role of Hydrogels in Propagating Pathogenic Molecules Including A*β* Oligomers, Tau, Exosomes, and Other Large Pathogens

**Figure 3:**
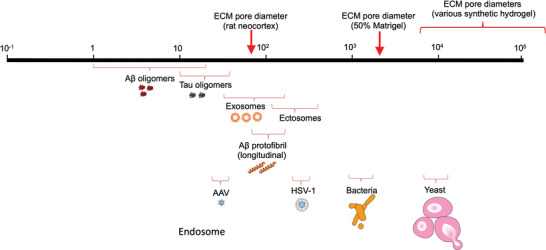
Average sizes of pathogenic molecules relevant to AD and ADRD. Pathogenic molecules, aggregates and microbes occur in a wide range of different sizes. The average pore diameter of a given matrix is a major determinant of the diffusivity of a particle, and thus impacts the spread or local retention of a pathogen. The pathogen illustrations are licensed from Motifolio Inc.

Several neurodegenerative disorders have recently been associated with the intercellular exchange, or spread, of pathogenic protein aggregates. Analogously, viruses and other monocellular pathogens move between cells in the CNS. Both spreading mechanisms require that relatively large particles pass through the extracellular space and thus interact with the ECM (**Figure** **3**). However, structures like exosomes fulfill important physiological roles in intercellular communication and cargo transport. These fundamentally beneficial processes can be impaired by pathogenic matrix alterations or hijacked to transport toxic materials.

A*β* oligomers in AD brains range in size from 1 to 20 nm.^[^ ^136^, ^137^, ^138^ ^]^ A*β* monomers (0.9 nm) and lower‐order amyloid aggregates can therefore be expected to spread relatively freely across the brain parenchyma, whereas high molecular weight A*β* oligomers, protofibrils, and fibrils (60–200 nm longitudinally) would be locally restricted.^[^ ^138^, ^139^ ^]^ This finding is most likely relevant to the various amyloid seeding effects via soluble oligomers found by, among others, the Jucker lab following injection of patient‐derived amyloid material into A*β* overproducing mouse brains.^[^ ^140^, ^141^ ^]^

In addition to A*β* oligomers, 3D ECM cell conditions would also impact spreading and seeding of oligomeric tau species. Animal models suggest that pathogenic tau species travel across synaptic connections in an activity‐dependent manner.^[^ ^142^, ^143^, ^144^, ^145^ ^]^ Therefore, elevated neuronal connections in the 3D culture system could augment the transfer of pathogenic tau species. Moreover, studies showed that soluble pathogenic tau species could directly seed further aggregation in other cells.^[^ ^146^ ^]^ Indeed, adding exogenous soluble tau aggregates is sufficient to induce tau aggregation and hyperphosphorylation in 3D‐cultured human iPSC‐derived cortical neurons.^[^ ^147^ ^]^ The limited diffusivity of tau species in the 3D matrix may also contribute to accumulating pathogenic soluble tau species, similar to A*β*.

Pathogenic protein aggregates inside cells can be also transmitted via synapses (direct contact), exosomes (30–150 nm) and ectosomes (100–350 nm), or through tunneling nanotubes (TNTs), rather than by diffusion.^[^ ^148^, ^149^, ^150^, ^151^, ^152^, ^153^ ^]^ In the rat neocortex, the average ECM pore diameter has been calculated as 38–64 nm, based on the diffusion rate of labeled nanoparticles after local injection.^[^ ^154^ ^]^ Magnetic resonance imaging (MRI) diffusion analyses in the brains of human subjects yielded matching results.^[^ ^155^ ^]^ Therefore, the presence of brain ECM, as well as a 3D culture system, would significantly limit diffusion of particles with larger diameters.

Viral infections illustrate the spread of nanoscale particles in the CNS. On the one hand, various viral families with differentially sized capsids can infect neural cells in a nonspecialized manner, e.g., lentivirus, adenovirus, adeno‐associated virus. On the other hand, neurotropic viruses have evolved to thrive in cells of the nervous system and thus show various optimizations for this tissue, e.g., herpes simplex virus or rabies virus. Conventional viral infection experiments via brain injection, e.g., for cell labeling or gene therapy, have repeatedly shown that viral spread is extremely limited in the brain parenchyma. Non‐neurotropic viruses only achieve highly localized infections. Notably, adeno‐associated virus (AAV) is both the smallest and most widely spreading and is used in most clinical trials for viral gene delivery to the CNS.^[^ ^156^ ^]^ AAVs are tiny particles with a diameter of 20 nm, smaller than exosomes (50–100 nm), which makes it suitable as a vector for brain gene delivery.^[^ ^157^, ^158^, ^159^ ^]^ In contrast, the neurotropic viruses (e.g., HSV‐1, approx. 170 nm) evolved techniques to travel along the microtubule network inside infected neurons and infect neighboring cells across synapses.^[^ ^160^ ^]^ Studies on synthetic particle motility in the rat brain have further confirmed that the ECM is responsible for the inefficient diffusion since digestion of hyaluronic acid and osmotic dilation of ECM pores drastically improved the distribution of 54 nm polymer nanoparticles from the injection site.^[^ ^161^ ^]^ Together, these results clearly support that the ECM play a critical role for viral infections in brains and 3D ECM cellular models.

Similarly, intracellular toxic protein aggregates (e.g., tau) have been shown to be transmitted dominantly at sites of cell–cell contact, i.e., at synapses, via exosomes and ectosomes, or through tunneling nanotubes (TNTs), rather than by diffusion.^[^ ^149^, ^151^, ^152^, ^153^ ^]^ In analogy to amyloid fibrils, mature tau fibrils that show paired helical filament (PHF) morphology are at least several hundred nanometers long, as one full turn of the filament spans 80–130 nm.^[^ ^162^, ^163^ ^]^ Yet, hyperphosphorylated, amorphous oligomeric tau has been shown to coexist with PHF tau in AD brains.^[^ ^164^ ^]^ These 10–30 nm oligomers are internalized by neural cells and cause fibrillation of normal tau and a breakdown of microtubules.^[^ ^165^, ^166^, ^167^ ^]^

We used 3D Matrigel with different concentrations for building 3D AD cellular models (9–50% final concentration).^[^ ^126^ ^]^ The stiffness and pore size varies depending on Matrigel concentration. The average pore size of 50% Matrigel is around 2 µm. Since decreasing Matrigel concentration would increase average pore sizes while decreasing gel stiffness,^[^ ^72^ ^]^ our 3D culture models provide full permeability for both small and large molecular pathogens implicated in AD. However, the presence of a compacted neural network inside Matrigel pores may limit diffusion of large particles. Indeed, we observed that lentiviral (80–120 nm) infection efficiency dramatically decreased in 3D human neural cell culture models as compared to the same cells differentiated in 2D conditions (our unpublished observation).

In summary, it is critical to choose the proper 3D ECM gels that mimic the physical property of natural brain ECMs to comprehensively recapitulate the propagation and aggregation of pathogenic molecules in AD. Currently, various hydrogels with ECM‐like properties are available, but the question of whether they provide an authentic micro topology is still unanswered. To illustrate: In stark contrast to the nanoscale pores in natural ECM, data on hydrogel matrices indicate that the pores are larger by several orders of magnitude, ranging from 3 to 600 µm depending on the material and fabrication technique (reviewed^[^ ^29^, ^168^ ^]^). However, these measurements stem from pure and “empty” hydrogels. In 3D matrix cell culture models, cell bodies and neurites fill the pores and secrete endogenous matrix components. To our knowledge, no data on the effective pore size of colonized hydrogel matrices are currently available. Still, hydrogel matrices restrict pathogenic particle diffusion and accumulation in 3D matrix cell culture systems, like the natural ECM in brains.^[^ ^9^, ^10^, ^11^, ^169^ ^]^ To better understand cell–matrix interactions in hydrogels, it is therefore warranted to measure pore sizes and other topological parameters of various hydrogels under these “tissue‐like” conditions

## 7 The Impacts of Aging on Brain ECM Stiffness and Composition on AD Pathogenesis

Aging a major risk factor for AD. According to the A*β* cascade hypothesis, delayed A*β* degradation in aged brain exacerbates A*β* accumulation in sporadic AD patients.^[^ ^6^ ^]^ The diffusion and local accumulation/aggregation of A*β* and other soluble pathogenic molecules in AD would be regulated by intrinsic properties of the ECM (e.g., average pore size and stiffness) and the fluidic equilibrium among blood plasma, CSF, ISF, and brain parenchyma. Indeed, aging is a critical factor altering brain ECM properties.^[^ ^170^ ^]^ In mice, aging increases brain ECM stiffness over time, and such stiffening can contribute to age‐associated pathology.^[^ ^34^ ^]^ This effect is hypothesized to depend on crosslinking of long‐lived matrix molecules, such as collagen I, with reactive glucose species (glycation), leading to increased matrix stiffness and reduced pore size.^[^ ^42^ ^]^ Accumulating advanced glycation end products (AGEs) have been associated with loss of tissue structure and elasticity in skin, bone, intestine, kidney, muscle, and brain (reviewed^[^ ^171^ ^]^).

However, on a macroscopic level, magnetic resonance elastography demonstrated a softening across various regions with progressing age in human brains.^[^ ^172^, ^173^ ^]^ Loss of macroscopic brain stiffness might reflect decreasing cell numbers and reduced neuronal network connectivity rather than ECM alterations. The cellular component makes up ≈70–80% of the brain matter versus 15–20% ECM and interstitial fluid, so alterations in cell density, connectivity and composition can easily overshadow subtle changes in the ECM (reviewed^[^ ^174^ ^]^).

While tissue stiffness decreases during normal aging, several studies demonstrated an exacerbated stiffness loss in AD compared to cognitively normal controls with and without amyloid deposits, as well as mild cognitive impairment.^[^ ^173^, ^175^ ^]^ As discussed earlier, the age‐ and AD‐associated ECM property alterations possibly contribute to the spread, accumulation, and aggregation of AD pathogenic molecules including soluble A*β* species. Age‐related changes in neuronal ECM also seem to be essential for the production and neurotoxicity of A*β*.^[^ ^176^, ^177^, ^178^, ^179^ ^]^ McKee and colleagues demonstrated that A*β* triggers a neurotoxic cascade in aging primates’ brains, whereas no effect was triggered in young primates’ brains.^[^ ^180^ ^]^ This might be connected to the large influence of matrix stiffness on brain cell differentiation and maintenance.^[^ ^181^ ^]^ Segel and colleagues demonstrated that age‐related dysfunction of oligodendrocyte precursor cells (OPCs) could be fully remedied by transplantation into a soft, youthful niche indicating that a “younger” matrix might enhance cellular resilience to other damage.^[^ ^34^ ^]^

Besides physical alterations, the brain interstitium undergoes specific compositional and biochemical changes in AD patients. Lepelletier and colleagues reported that the ECM proteins collagen IV, fibronectin, and perlecan appear enriched in early and late AD, yet none colocalized with amyloid plaques.^[^ ^182^ ^]^ In contrast, both the chondroitin and the heparan sulfate families of proteoglycans (CSPGs and HSPGs) have been shown to colocalize with amyloid plaques in human AD and Down syndrome patient brain tissue.^[^ ^183^, ^184^, ^185^ ^]^ In addition, CSPGs and HSPGs have been detected inside the cytoplasm of a subset of neurons and astrocytes in AD.^[^ ^186^, ^187^ ^]^ Therefore, it was hypothesized that HSPGs promote amyloid deposition and/or prevent efficient clearance. Notably, several HSPGs have been found enriched in the brains of AD patients, including syndecan‐3, syndecan‐4, glypican‐1, glypican‐3, and perlecan. It is not yet clear which one is responsible for the amyloid clearance impairment, or whether there is a functional difference at all with regard to the amyloid pathology (see refs. [188, 189] for a more detailed discussion of the various proteoglycans in AD, see ref. [181]).

The extracellular matrix also plays a pivotal role in intercellular communication. To give one example, knockout experiments have shown that the ECM protein tenascin C is required for astrocyte generation in the mouse ventral spinal cord via tuning of neural precursor cell sensitivity to EGF and FGF.^[^ ^190^ ^]^ If – in addition to tenascin C – tenascin R, brevican, and neurecan are knocked out, mouse cortical neurons generate more excitatory synapses than usual, likely via disruption of peri‐neuronal nets that are typically formed from neuronal, astrocytic, and oligodendrocytic proteins.^[^ ^191^ ^]^ Also, tenascin C is deposited around neuritic but not diffuse plaques in the cortices of AD patients and cognitively normal subjects with amyloid plaque pathology, but not in plaque‐free individuals.^[^ ^192^ ^]^ While the function of plaque‐associated tenascin C is not yet known, these interactions underline the multifunctionality of even single ECM proteins.

The aged ECM could also alter migratory behavior of innate immune cells. We recently presented a 3D “tri‐culture” model of AD (neurons, astrocytes, microglia), showing that microglia in 3D Matrigel cultures can sense APP‐driven pathology remotely and react by migrating toward and through the 3D Matrigel, interacting with amyloid deposits and the affected neural cells.^[^ ^169^ ^]^ Such migratory behavior can be modulated by ECM components. For example, chondroitin sulphate proteoglycans (CSPGs) are deposited in the vicinity of glial scars where they cause growth‐cone collapse and block migration via RhoA/ROCK signaling.^[^ ^193^, ^194^ ^]^ Analogously, if less dramatically, common ECM proteins like laminin, fibronectin, and vitronectin differentially affect microglial activation and expression of migration‐associated integrin receptors in vitro.^[^ ^195^ ^]^

Additionally, the blood–brain barrier (BBB) undergoes progressive breakdown during aging.^[^ ^196^ ^]^ The BBB consists of endothelial cells, pericytes, astrocytes, and neurons, as well as a basement membrane secreted by pericytes.^[^ ^197^ ^]^ Physiologically, it excludes most cells and molecules from entering the brain through highly restrictive tight junctions between endothelial cells and limited transcytosis.^[^ ^197^ ^]^ As the BBB ages, expression of tight junction proteins like ZO‐1 and claudin‐5 lessens, and transcytotic transport mechanisms slow down. Albumin, K^+^ ions, glutamate, antibodies, and immune cells can then enter the brain and cause excitotoxicity and inflammation. BBB breakdown is also a well‐known part of AD pathology and can be used as a diagnostic biomarker.^[^ ^198^, ^199^, ^200^ ^]^

While there are still more questions than answers regarding cell–cell and cell‐ECM crosstalk in a complex, tissue‐like context, it is critical to establish viable model systems to explore those interactions. Multiple 3D BBB models have been reported, which recapitulate key aspects of BBB dysfunction observed in AD patients.^[^ ^13^, ^201^ ^]^ However, these models do not fully recapitulate comprehensive BBB structures or BBB aging.

## 8 Improved 3D ECM Systems for Modeling AD and the Aged ECM in a Dish

**Figure 4:**
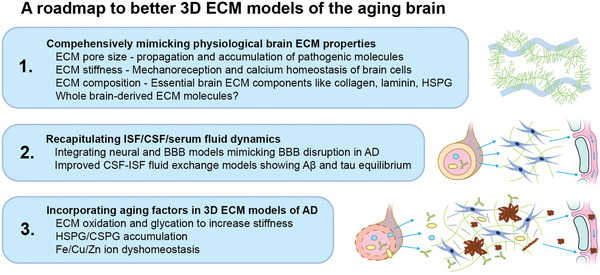
Building an improved 3D ECM model for AD. Insights from studies of ECM composition, interstitial fluid composition and dynamics, blood–brain barrier leakage, reaction space confinement, and aging can contribute to a more authentic recapitulation of the extracellular space in future models of AD and other age‐related extracellular proteinopathies.

This review underscores the critical role of brain ECM in aging and AD pathogenesis through multiple, as of yet elusive mechanisms. Most current 3D AD cellular models depend on artificial ECM‐like gels with under‐characterized properties. However, emerging 3D matrix cell culture models with varied ECM properties could provide an exciting opportunity to close the knowledge gap between brain ECM aging and AD pathogenesis. Currently, no available 3D cellular models address the impact of ECM aging on the pathogenic cascade of AD. Here, we propose to address the following considerations to explore interactions among ECM, aging, and AD in cellular models (**Figure** **4**).

First, physical matrix properties need to mimic physiological brain ECM properties. Specifically, a given matrix should be adjusted for the optimal pore size and stiffness comparable to brain ECMs. On the one hand, defined pore size is vital to control solute diffusion, confined biocrystallization processes likely underlying amyloid aggregation, as well as the spread of pathogenic molecules. On the other hand, matrix stiffness impacts cellular mechanoreception, fate decisions and Ca^2+^ homeostasis. Candidate matrices with physiological stiffness and pore size have been systematically reviewed before.^[^ ^29^, ^50^, ^168^ ^]^ Recreating an entirely artificial, authentic ECM is not yet possible. But a good approximation would cover all main classes of ECM molecules carrying the relevant post‐translational modifications in a defined and tunable fashion. Matrigel's heterogeneity and poor characterization make it an unsuitable starting point for creating such a matrix. In contrast, human brain‐derived neural matrices might bridge the gap until a fully synthetic alternative is available. But the feasibility of recapitulating local brain ECM composition from mixed, decellularized brain fractions, as well as issues of availability and heterogeneity have not been fully addressed.

Second, extracellular fluid dynamics must mirror physiological parameters to account for build‐up and wash‐out effects of paracrine factors and pathogenic molecules as observed in conventional tissue culture. This issue is caused by fluid exchange between the relatively large volume of cell culture media and cell compartments embedded inside ECM‐like gel. Recapitulating fluid equilibrium between blood plasma, CSF, ISF, and brain parenchyma in a dish is challenging. The first steps in that direction were taken via microfluidic devices designed to mimic brain ISF flow^[^ ^202^ ^]^ and blood–brain‐barrier (BBB) function.^[^ ^203^, ^204^ ^]^ Later, Maoz et al. introduced a complicated microfluidic organ chip to model influx/efflux across BBB and neurons.^[^ ^205^ ^]^ In parallel, Pellegrini et al. reported a brain organoid model mimicking CSF production by the choroid plexus to predict brain permeability of new compounds.^[^ ^206^ ^]^ Combining these models might allow capturing authentic fluid dynamics and enable the investigation of pathogenic molecule equilibrium states, e.g., in the context of intact versus compromised BBB structures.

Third, aging of the extracellular milieu can be modeled by modulating matrix and fluid parameters. Matrix stiffness is positively correlated with age and age‐related tissue dysfunction, so adjusting stiffness to the high end of physiological levels or beyond could aggravate pathogenic phenotypes.^[^ ^34^ ^]^ It is possible that replicating other age‐associated features like specific ECM crosslinks, e.g., via oxidation and glycation, will add to phenotype severity.^[^ ^42^ ^]^ Select ECM component proteins are specifically associated with A*β* retention and aggregation in brains, and therefore it is important to fully characterize the amounts and ratios of these molecules in AD brains and 3D cellular models. HSPGs like syndecan‐3, syndecan‐4, glypican‐1, glypican‐3, or perlecan likely inhibit monomer wash‐out during medium changes.^[^ ^188^, ^189^ ^]^ Some brain ECM proteins have been shown to decrease amyloid deposition and A*β* fibril formation in vitro, especially laminin.^[^ ^207^, ^208^, ^209^ ^]^ Simpson and colleagues recently reported that high contents of laminin in Matrigel (≈60%) prevents A*β* fibril formation in vitro.^[^ ^210^ ^]^ However, since Matrigel has been used successfully in AD models, the high laminin content might be counteracted by other components like HSPGs.^[^ ^10^ ^]^

Extracellular fluid dynamics are impacted in aging as the BBB becomes leaky and serum components as well as immune cells can enter the brain parenchyma. We previously reported a microfluidic 3D AD culture model of the A*β* equilibrium across an artificial BBB composed of human endothelial cells.^[^ ^13^ ^]^ Interestingly, the accumulation of pathogenic A*β* species at the endothelial cells in this model disrupted BBB function in this system similarly to the BBB deficits in AD brains. Recently, Blanchard et al. presented a human iPSC‐derived self‐assembling brain BBB model using 3D Matrigel to interrogate the role of APOE4 in BBB A*β* accumulation.^[^ ^201^ ^]^ Furthermore, medium additives like serum, APOE4 or metal ions (e.g., copper, iron, or zinc) were shown to support pathogenesis in a 3D ECM.^[^ ^211^ ^]^ However, it might be preferable to rethink current culture media, and to design new synthetic interstitial fluids with the express goal of promoting biocrystallization. Such a novel mimetic fluid would enable the study of biocrystallization, its dependence on matrix‐mediated confinement, and the influence of various matrix parameters along the stages of AD pathology.

## 9 Conclusions

The extracellular environment is an essential and often overlooked component of tissue‐level effects, such as the accumulation and spread of pathogenic protein aggregates. Due to its innate complexity, it is still challenging to understand its functional roles comprehensively and to implement and standardize in vitro models of human diseases. As we discussed in the context of the nervous system and specifically Alzheimer's disease, the extracellular matrix is a key component of the A*β* and tau pathologies on multiple organizational levels. Matrix molecules can recruit pathogenic proteins by direct interactions, leading to local supersaturation and promoting the spread of pathologies between cells. The pore size and macromolecular crowders confine solutes and drastically increase effective concentration. Pore size affects the motility of larger particles like aggregates and exosomes, potentially locking in pathogenic material. The extracellular fluid also contributes to the equilibrium and degradation of pathogenic molecules among brain cells and ECM. It is expected to strongly influence aggregation processes via ionic strength, solute exclusion, and complex cocrystallization. Aging, a major risk factor for most neurodegenerative diseases, alters the physical properties of brain ECM and its composition, which would play roles in the accumulation and propagation of pathogenic molecules associated with brain diseases. Substantial research will be required to build a solid understanding of these components and their interplay to develop better models of the aged and diseased brain in a dish.

## Conflict of Interest

The authors declare no conflict of interest.
